# Amido-bridged nucleic acid (AmNA)-modified antisense oligonucleotides targeting α-synuclein as a novel therapy for Parkinson’s disease

**DOI:** 10.1038/s41598-019-43772-9

**Published:** 2019-05-21

**Authors:** Takuya Uehara, Chi-Jing Choong, Masayuki Nakamori, Hideki Hayakawa, Kumiko Nishiyama, Yuuya Kasahara, Kousuke Baba, Tetsuya Nagata, Takanori Yokota, Hiroshi Tsuda, Satoshi Obika, Hideki Mochizuki

**Affiliations:** 1 Graduate School of Medicine, Osaka University, Suita, Japan; 2 National Institutes of Biomedical Innovation, Health and Nutrition, Suita, Japan; 3 Graduate School, Tokyo Medical and Dental University, Tokyo, Japan; 4 Graduate School of Pharmaceutical Sciences, Osaka University, Suita, Japan

**Keywords:** RNAi, Parkinson's disease

## Abstract

Parkinson’s disease (PD) is a neurodegenerative disease caused by the loss of dopaminergic neurons in the substantia nigra. A characteristic pathological feature of PD is cytoplasmic accumulation of α-synuclein (SNCA) protein. Multiplication of the *SNCA* gene in familial PD and pathological accumulation of SNCA protein during progression of sporadic PD suggest that increased SNCA protein levels increase the risk of PD. Thus, reducing SNCA expression levels could delay PD onset or modify the disease course. For efficient knock down, we designed and synthesized an amido-bridged nucleic acids (AmNA)-modified antisense oligonucleotide (ASO) that targeted SNCA with improved stability and cellular uptake *in vivo*. AmNA-ASO efficiently downregulated SNCA at both the mRNA and protein level *in vitro* and *in vivo*. Notably, AmNA-ASO was efficiently delivered into the mouse brain by intracerebroventricular injection without the aid of additional chemicals. Furthermore, administration of AmNA-ASO ameliorated neurological defects in PD model mice expressing human wild type SNCA. Taken together, these findings suggest that AmNA-ASO is a promising therapeutic strategy for SNCA-associated pathology in PD.

## Introduction

Parkinson’s disease (PD) is a neurodegenerative disease caused by the loss of dopaminergic neurons in the nigrostriatal pathway^1^. Clinical manifestations of PD include bradykinesia, muscular rigidity, resting tremors, and postural instability. Although replacement therapy with the dopamine precursor, levodopa, is effective for these symptoms of PD^2^, chronic treatment is associated with the development of motor complications and the disease is inexorably progressive^3,4^—possibly because degeneration of nigrostriatal neurons causes progressive dopamine loss.

α-synuclein (SNCA) protein is a major component of the Lewy body, a pathological hallmark of both the sporadic and familial forms of PD^5,6^, which suggests that dysfunction or toxicity caused by SNCA protein results in the pathology of PD. A missense mutation in the *SNCA* gene was first identified in PARK1, a dominant familial form of PD^7^. In addition, duplication or triplication of the *SNCA* gene has been shown to cause the dominant form of PD, PARK4^8,9^. Polymorphisms in the *SNCA* gene are also associated with susceptibility to sporadic PD and tend to be correlated with SNCA mRNA levels^10^. Our group and other researchers have shown that wild type SNCA causes cell-autonomous toxicity when expressed specifically in nigral dopaminergic neurons^11–14^. Because SNCA pathology extends from the spinal cord, brainstem, or olfactory bulb to the cortex during PD progression^15–18^, decreasing SNCA expression levels could be an attractive treatment for suppressing PD.

The antisense oligonucleotide (ASO) is a potential gene therapy for targeting the *SNCA* gene. ASO-based therapies have already been approved for neuromuscular diseases including spinal muscular atrophy (SMA) and Duchenne muscular dystrophy^19–22^.

The nucleic acids of ASO can be modified to acquire high nuclease resistance and efficient binding affinities toward complementary strands^23,24^. We have shown that ASOs with amido-bridged nucleic acid (AmNA), an analog of locked nucleic acid (LNA) with modification of the amide bond bridged between the 2′ and 4′ carbons of the ribose, show higher knockdown efficiency and safety compared to natural ASO and LNA^25–27^ **(**Supplemental Fig. 1a**)**.

In the present study, our designed AmNA-ASO significantly reduced human SNCA (hSNCA) mRNA and protein levels in human cultured cells and in mice. After administering AmNA-ASO into the intracerebroventricular space of the mouse brain, AmNA-ASO was widely distributed throughout the brain and efficiently taken up by neuronal and, to a lesser extent, non-neuronal cells without the aid of additional chemicals. Moreover, a single injection of AmNA-ASO ameliorated the defects observed in transgenic mice expressing wild type SNCA. Hence, this work highlights the potential of SNCA-targeted AmNA-ASO therapy for PD.

## Results

### AmNA-ASO efficiently reduced SNCA mRNA levels in human cultured cells

To determine the potency of AmNA-ASO for reducing the levels of SNCA mRNA, we generated a series of AmNA-ASOs (n = 50) covering 80.7% of the coding sequence of SNCA mRNA. The AmNA-ASOs were designed to be 15-mer chimeric antisense oligonucleotides (gapmer) containing AmNA. Supplemental Fig. 1b illustrates the designed AmNA-ASO, which contains AmNA at each end flanking the central bases of DNA with a gapmer motif of 3AmNA-9DNA-2AmNA-1DNA (3-9-2-1).

**Figure 1:**
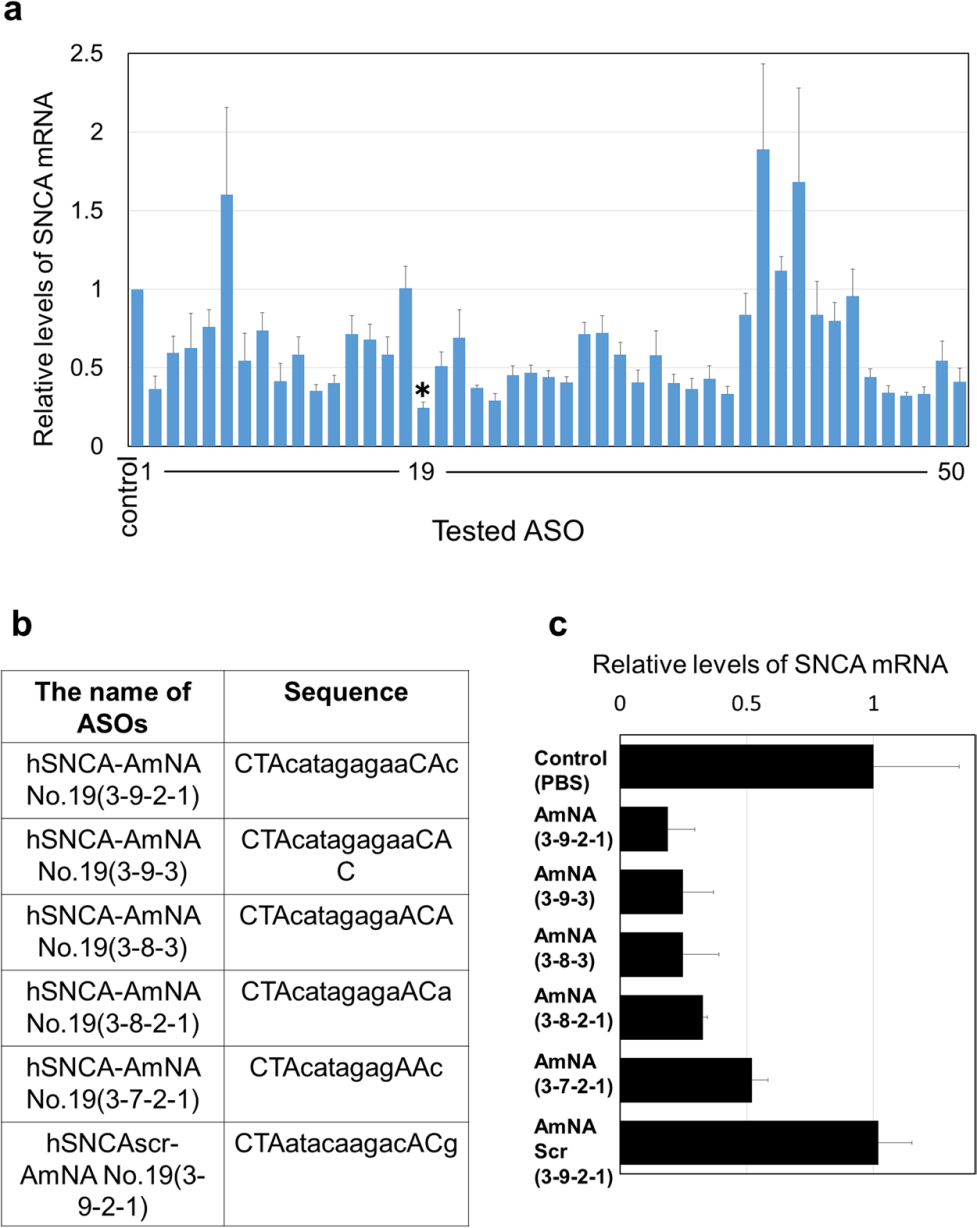
ASO^A19^ (3-9-2-1) efficiently downregulated the level of SNCA mRNA in HEK293 cells. (**a**) qPCR analysis showing the expression levels of SNCA mRNA in HEK293 cells. A total of 50 AmNA-ASOs were individually transfected into HEK293 cells. ASO^A19^ significantly reduced levels of hSNCA mRNA. Data are expressed as mean ± SEM (n = 6). **p < 0.01 by Dunnett’s test. (**b**) Sequences of ASO^A19^ containing various gapmer motifs. Capital and small letters represent AmNA and DNA, respectively. (**c**) Assessment of ASO^A19^ with variable gapmer motifs. qPCR analysis showing the expression levels of SNCA mRNA in HEK293 cells. ASO^A19^ (3-9-2-1) reduced SNCA mRNA more efficiently than the other modified ASO^A19^. Data are expressed as mean ± SEM (n = 3).

We screened synthesized AmNA-ASOs for knockdown efficiency of SNCA mRNA in human embryonic kidney 293 (HEK293) cells that express hSNCA mRNA endogenously. For screening, we transfected AmNA-ASOs into HEK293 cells at a single concentration (50 nM) and quantified the SNCA mRNA level using quantitative polymerase chain reaction (qPCR) 24 hours after transfection. We found that several AmNA-ASOs significantly reduced SNCA mRNA levels. In particular, AmNA-ASO No.19 significantly decreased the SNCA mRNA level to 24.5% of the normal expression level in mock transfected cells (control = 100% ± 8.52%, AmNA-ASO No.19 = 24.5% ± 2.29%, p < 0.01 by Dunnett’s test), suggesting that AmNA-ASO No. 19 is highly potent for targeting SNCA mRNA in human cultured cells **(**Fig. 1a**)**. To determine the most efficient construct of AmNA-ASO No. 19, we generated AmNA-ASOs No. 19 with variable lengths and gapmer motif modifications. As a control, we used phosphate-buffered saline (PBS) and scr-AmNA containing the same base composition as AmNA-ASOs No. 19 but in a scrambled order **(**Fig. 1b**)**. We then transfected the ASOs into HEK293 cells and measured the level of SNCA mRNA expression by qPCR 24 hours after transfection. We found that all of the tested AmNA-ASOs significantly reduced the SNCA mRNA level. AmNA-ASO No. 19 with the gapmer motif of 3AmNA-9DNA-2AmNA-1DNA (3-9-2-1), the same sequence used for the screening described above, was the most efficient and downregulated the SNCA mRNA level to 19.0%, suggesting that AmNA-ASO No. 19 3-9-2-1 (hereinafter referred to as ASO^A19^) is highly potent for targeting SNCA mRNA in the human cultured cells **(**Fig. 1c**)**.

**Figure 2:**
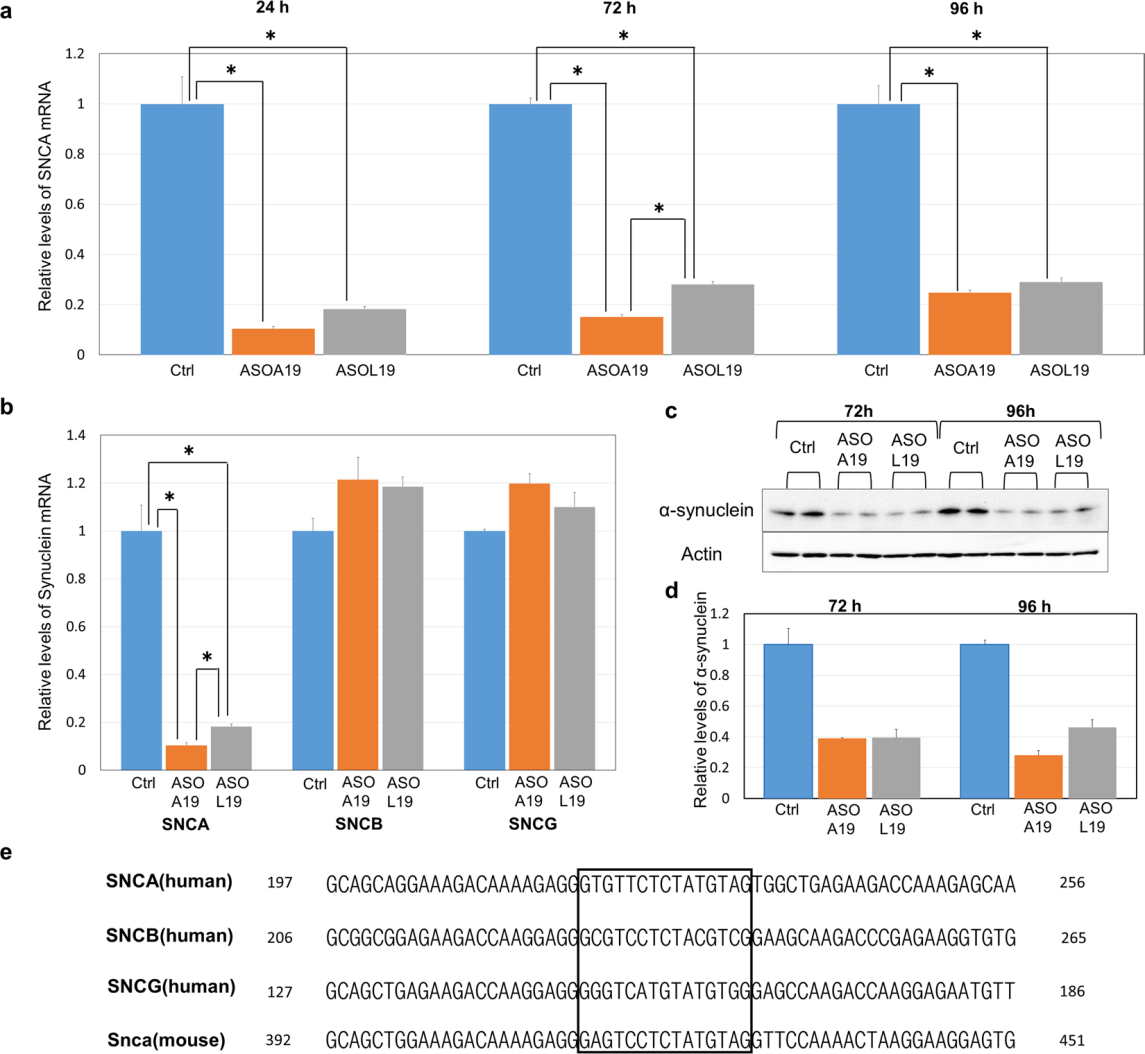
ASO^A19^ was more efficient than ASO^L19^ in HEK293 cells. (**a**) qPCR analysis showing SNCA mRNA in HEK293 cells transfected with ASO^A19^ and ASO^L19^. ASO^A19^ decreased the expression levels of SNCA mRNA compared to ASO^L19^ 24 and 72 hours after transfection. Data are expressed as mean ± SEM (n = 3). *p < 0.01 by one-way ANOVA, Tukey post hoc test. (**b**) qPCR analysis of SNCA, SNCB, and SNCG mRNA in HEK293 cells transfected with ASO^A19^ and ASO^L19^. Although ASO^A19^ decreased the expression levels of SNCA mRNA (see a for comparison), SNCB and SNCG levels showed no reduction. *p < 0.01 by one-way ANOVA, Tukey post hoc test. (**c**,**d**) Immunoblot and densitometry analysis showing that both ASO^A19^ and ASO^L19^ reduced SNCA protein levels. Proteins were extracted from HEK293 cells 72 and 96 hours after transfection of ASO^A19^ and ASO^L19^ and subjected to immunobloting. Images of blots were cropped from different parts of the same gel. Full-length gels are shown in Supplemental Fig. 2. Immunoblotting with anti-beta-actin was used as an internal control. Densitometry data are expressed as mean ± SEM (n = 2). (**e**) Alignment and relationship of the base sequences of four: synuclein family, human SNCA, SNCB, SNCG, and mouse Snca. The square enclosed area represents the sequence that ASO^A19^ can target.

AmNA-ASOs were shown to be less toxic to animals than LNA-ASOs^27^. To compare the knockdown efficiencies of AmNA- and LNA-ASOs, we generated LNA-ASO No. 19 3-9-2-1 (hereinafter referred to as ASO^L19^) carrying the same target sequence as ASO^A19^. We transfected ASO^A19^ and ASO^L19^ into HEK293 cells and measured the levels of SNCA mRNA expression by qPCR 24, 72, and 96 hours after transfection, as described above. We found that ASO^A19^ reduced the SNCA mRNA levels to 10.3% (24 h) and 15.2% (72 h), whereas ASO^L19^ reduced the levels to 18.3% (24 h) and 28.1% (72 h), suggesting that ASO modified with AmNA knocked down SNCA mRNA more efficiently than that modified with LNA (p = 0.0076, one-way ANOVA, Tukey post hoc test) **(**Fig. 2a**)**. To evaluate the off-target effects of ASO^A19^, we measured the levels of β-synuclein (SNCB) and γ-synuclein (SNCG), which are members of the synuclein family. We found no significant difference in mRNA levels between control and ASO^A19^-treated cells, suggesting no off-target effects of ASO^A19^ on SNCB and SNCG (Fig. 2b). We also evaluated SNCA protein levels by Western blotting 24, 48, 72, and 96 hours after transfection. ASO^A19^ reduced the expression levels of SNCA protein to similar levels as ASO^L19^ at both 72 and 96 hours after transfection **(**Fig. 2c,d and Supplemental Fig. 2**)**, although protein levels were not yet reduced at 24 and 48 hours **(**Supplemental Fig. 3**)**. Taken together, these data indicate that ASO^A19^ can reduce SNCA mRNA and protein levels in human cultured cells and that the potency of ASO^A19^ is comparable to or more efficient than that of ASO^L19^.

### ASO^A19^ was efficiently delivered into mouse brains, unassisted by transfection reagents

**Figure 3:**
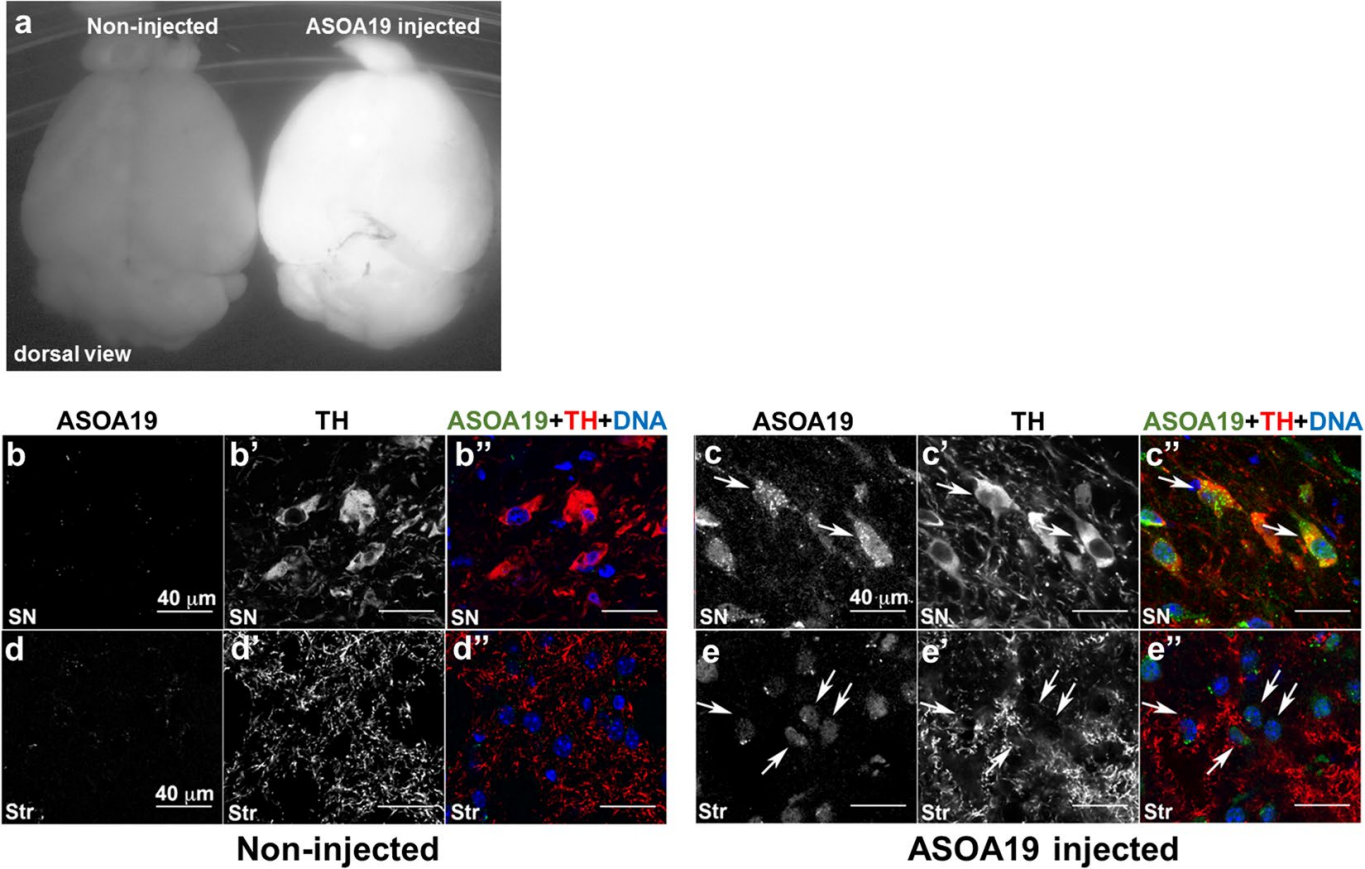
ASO^A19^ injected into the lateral ventricle was efficiently delivered throughout the mouse brain. (**a**) Auto-fluorescence image of the brain showing efficient delivery of ASO^A19^ by intracerebroventricular injection. Fluorophore-conjugated ASO^A19^ was widely distributed into brain 48 hours after injection into the left lateral ventricle. A non-injected brain (left) and brain injected with ASO^A19^ conjugated with Alexa 488 (right) are shown. (**b**–**e**) Uptake of ASO^A19^ by presynaptic and postsynaptic tyrosine hydroxylase (TH)-positive neurons. ASO^A19^ was localized in TH-positive neurons in the substantia nigra (SN) and distributed into neurons contacting TH immunoreactive axons in the striatum (Str) (arrow). Immunofluorescence images of the SN (b and c) and striatum (**d**,**e**). Auto-fluorescence of AmNA-Alexa488 (**b**–**e**) and immunofluorescence with TH (**b’**–**e’**). Merged images showing AmNA-Alexa488 (in green), TH (in red), and DAPI-labeled cell nuclei (in blue) (**b”**–**e”**).

**Figure 4:**
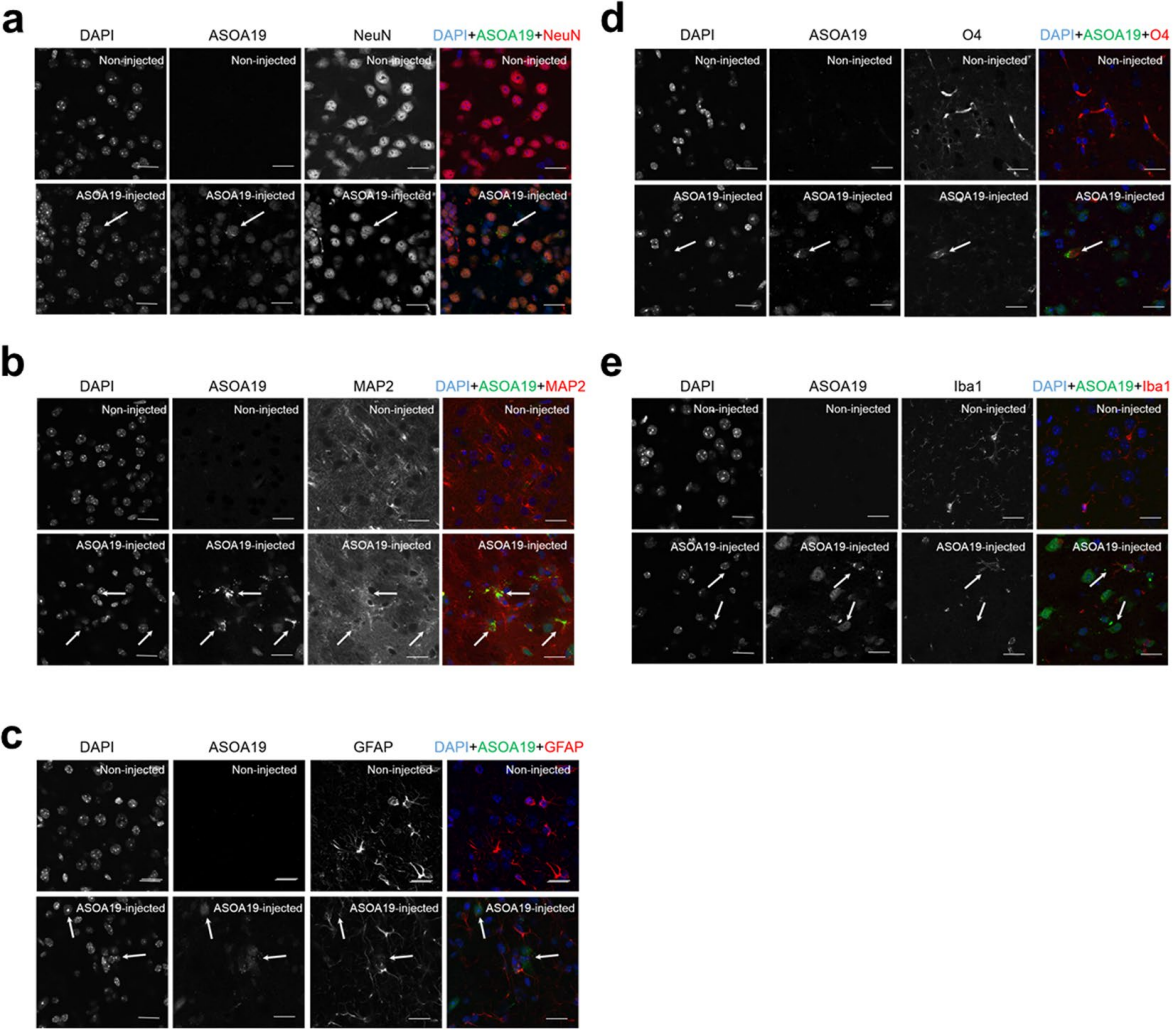
Uptake of ASO^A19^ by various cell types in the mouse brain. Immunostaining images of the (**a**) substantia nigra and (**b**–**e**) striatum of Alexa 488 conjugated-ASO^A19^-administered wild type mice. Uptake of ASO^A19^ by neurons, astrocytes, oligodendrocytes, and microglia was seen. ASO^A19^ was distributed into cells stained with NeuN (**a**), MAP2 (**b**), GFAP (**c**), O4 (**d**), and Iba1 (**e**) (arrow). Merged images showing AmNA-Alexa488 in green, NeuN (**a**), MAP2 (**b**), GFAP (**c**), O4 (**d**), Iba1 (**e**) in red, and DAPI-labeled cell nuclei in blue. Scale bar: 20 μm.

**Figure 5:**
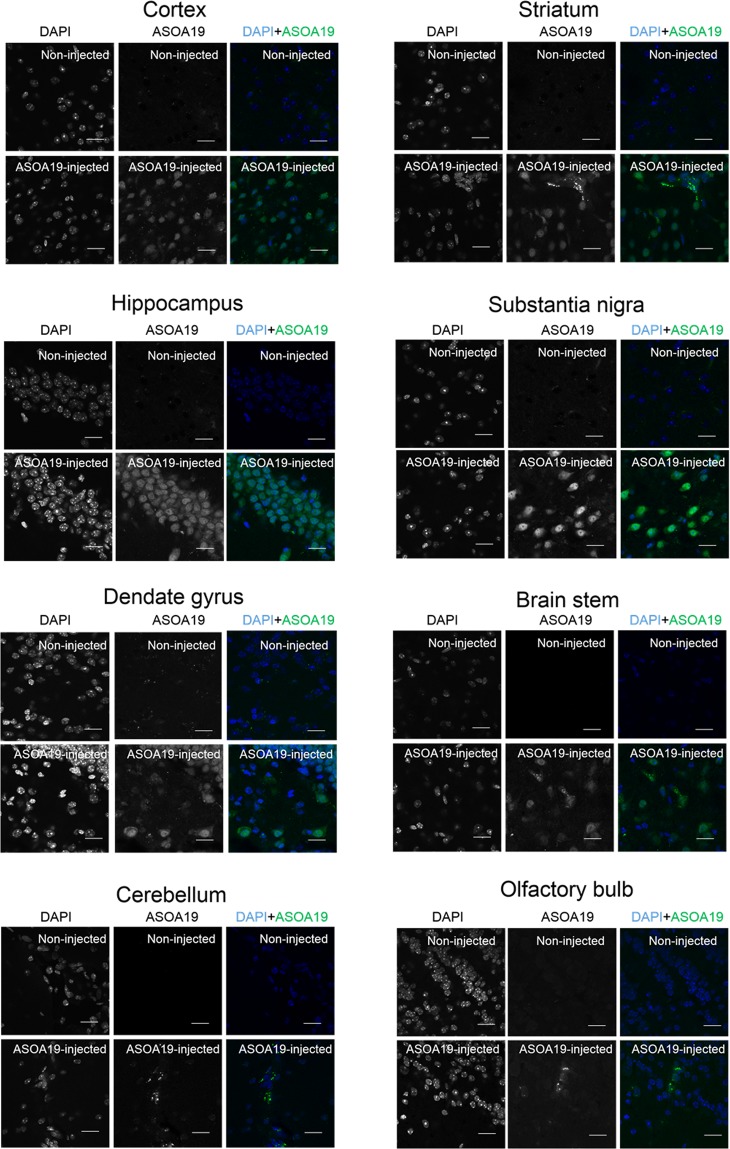
Widespread distribution of ASO^A19^ throughout the mouse brain. Immunostaining images of brain sections collected from Alexa 488 conjugated-ASO^A19^-administered wild type mice. Widespread distribution of ASO^A19^ in different brain areas, including the cortex, olfactory bulb, hippocampus, dentate gyrus, striatum, substantia nigra, cerebellum, and brain stem, was seen. Merged images showing AmNA-Alexa488 in green and DAPI-labeled cell nuclei in blue. Scale bar: 20 μm.

To establish a future ASO treatment for PD, we determined if ASO^A19^ was effective for targeting SNCA in PD model mice. Intrathecal administration of ASO has recently been established for the treatment of patients affected with SMA^20^. To determine if ASO^A19^ can be delivered from cerebrospinal fluid (CSF) space to neurons in the central nervous system (CNS), we injected ASO^A19^ conjugated with fluorescent Alexa 488 into the left lateral ventricle of mice. We extracted the mouse brains 48 hours after injection and examined the localization of ASO. Auto-fluorescence images showed that ASO^A19^ conjugated with Alexa 488 was widely distributed in the CNS after intracerebroventricular injection **(**Fig. 3a**)**. Immunofluorescence images showed that ASO^A19^ was localized in tyrosine hydroxylase (TH) positive neurons in the substantia nigra (arrow in Fig. 3c-c”**)** and neurons surrounded by TH-positive axons in the striatum (arrow in Fig. 3e-e”**)**, suggesting that ASO^A19^ could be efficiently delivered to TH- and non-TH-positive neurons *in vivo* (Fig. 3b–e**)**. To further characterize the cell types that take up ASO^A19^, we immunostained the mouse brain sections for NeuN and MAP2 (neuronal markers), GFAP (astrocytes marker), O4 (oligodendrocytes marker), and Iba1 (microglial marker). ASO^A19^ was mainly taken up by neuronal cells (arrow in Fig. 4a,b) and, to some extent, by oligodendrocytes, astrocytes, and microglial cells **(**arrow in Fig. 4c–e**)**. Furthermore, we evaluated the brain distribution of ASO^A19^ and found ASO^A19^ in various brain areas including cortex, olfactory bulb, hippocampus, dentate gyrus, striatum, substantia nigra, cerebellum, and brain stem **(**Fig. 5**)**. These results suggest that ASO injected into the left ventricle is distributed into various cell types and brain areas without the aid of chemical reagents.

### ASO^A19^ efficiently targeted SNCA in transgenic mice expressing hSNCA

**Figure 6:**
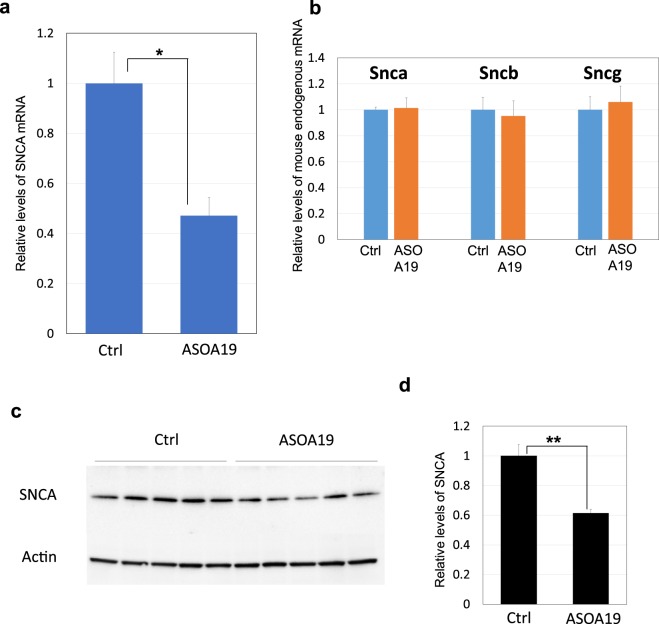
Intracerebroventricular administration of ASO^A19^ reduced SNCA mRNA and protein levels in brains of TH-SNCA mice. (**a**,**b**) qPCR analysis of mRNA levels of hSNCA and mouse endogenous Snca, Sncb, and Sncg in mouse brains. ASO^A19^ or control was injected into the left lateral ventricle of TH-SNCA-140 m mice expressing human SNCA with Ala53Thr mutation. Total RNA was extracted from left cerebral hemisphere samples collected 14 days after ASO^A19^ injection. ASO^A19^ significantly reduced the levels of SNCA mRNA. The levels of Snca, Sncb, and Sncg mRNA showed no reduction. Data are expressed as mean ± SEM (n = 5). *p < 0.05 by t-test. (**c**,**d**) Immunoblotting and densitometry analysis showing that ASO^A19^ reduced the levels of SNCA protein. Proteins were extracted from right cerebral hemisphere samples two weeks after administration of ASO^A19^ and subjected to immunoblotting. The images of blots were cropped from different parts of the same gel. Full-length gels are shown in Supplemental Fig. 4. Immunoblotting with anti-beta-actin was used as an internal control. ASO^A19^ significantly reduced the levels of SNCA protein. Densitometry data are expressed as mean ± SEM (n = 5).

To determine if ASO^A19^ injected into the ventricle could reduce hSNCA expression in the CNS, we tested its effects in the transgenic PD mouse model TH-SNCA-140 m. TH-SNCA-140 m mice carry the *hSNCA* gene with an Ala53Thr mutation, the expression of which is driven by the TH promoter^28^. We injected 100 μg of control ASO (ASO containing scrambled sequences) and ASO^A19^ into the left lateral ventricles of two-month-old TH-SNCA-140 m transgenic mice in a single application. Neither injection caused any toxicity-related changes in general health status or behavior. Two weeks after injection, we extracted mRNA from the left cerebral hemisphere and measured hSNCA mRNA levels. Quantification by qPCR showed that ASO^A19^ significantly decreased hSNCA mRNA levels relative to control ASO (100% (control) vs. 47.2% (ASO^A19^), p < 0.05, t-test, n = 5), which suggests that ASO^A19^ injected into the CSF can reduce hSNCA mRNA levels in the brain **(**Fig. 6a**)**. To evaluate the off-target effects of ASO^A19^, we measured the levels of endogenous α-synuclein (Snca), β-synuclein (Sncb), and γ-synuclein (Sncg) in mice. We found no significant differences in mRNA levels of these substances between control and ASO^A19^-treated mice, suggesting that ASO^A19^ has no off-target effects (Fig. 6b).

We also evaluated hSNCA protein levels in the right cerebral hemisphere by Western blotting two weeks after injection. ASO^A19^ significantly reduced the expression levels of SNCA protein (Fig. 6c,d and Supplemental Fig. 4). Taken together, these data indicate that ASO^A19^ can reduce SNCA mRNA and protein levels in the brains of TH-SNCA-140 m transgenic mice.

### AmNA-ASO ameliorated defects in motor behavior observed in hSNCA transgenic mice

To further confirm the efficacy of ASO^A19^, we tested it in another transgenic mouse line Thy1-SNCA, which carries human wild type *SNCA* driven by the murine thymus cell antigen 1 (Thy1) promoter. High levels of hSNCA expression are present in the neocortex, hippocampus, olfactory bulb, thalamus, colliculi, substantia nigra, and brainstem of Thy1-SNCA mice^29^. Moreover, Thy1-SNCA mice have a key feature of PD: high levels of SNCA phosphorylated at serine 129, which is shown to be the predominant form of SNCA found in Lewy bodies, in the substantia nigra, striatum, cortex, frontal cortex, and hippocampus^30^.

**Figure 7:**
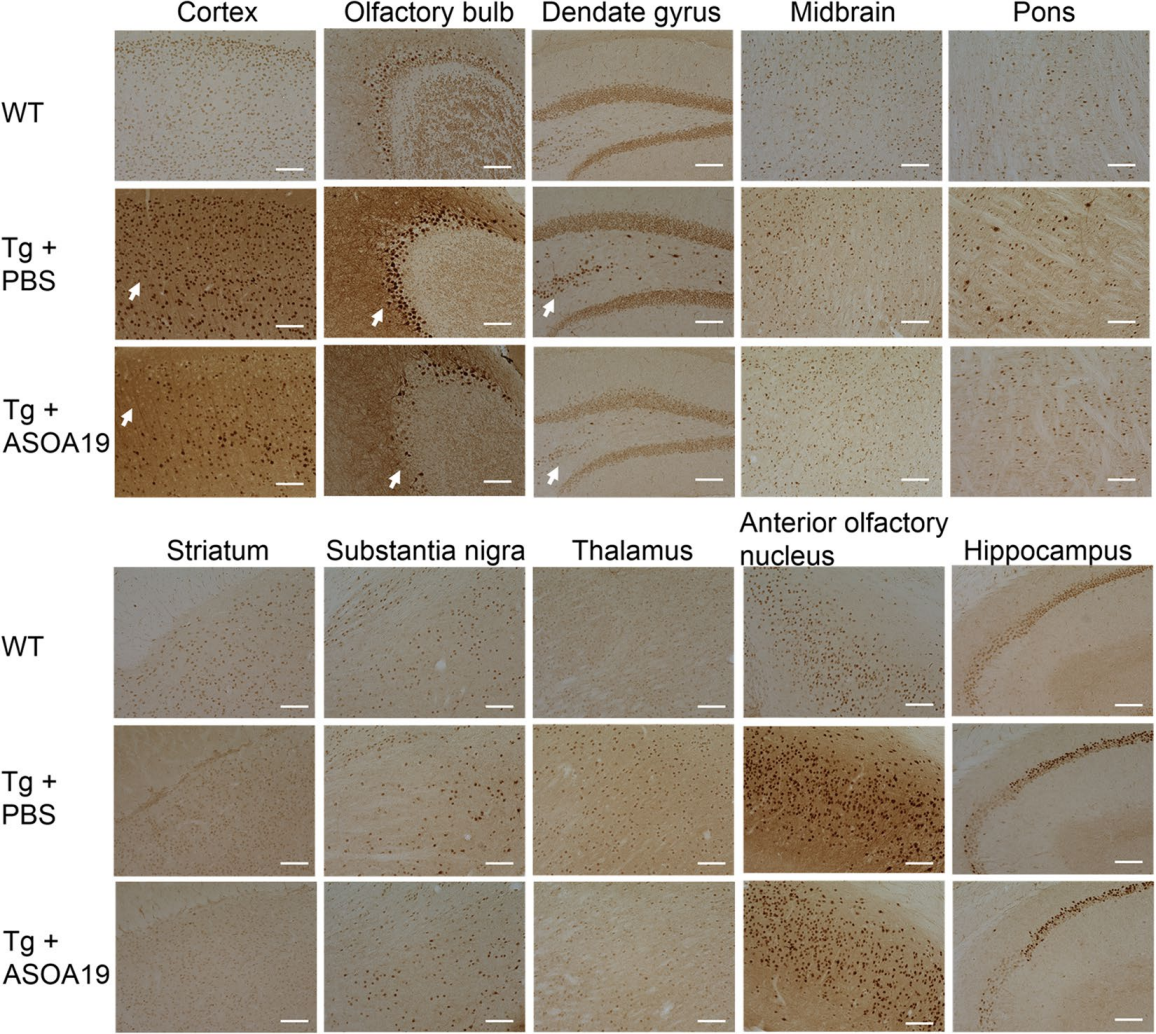
Decrease of phosphorylated SNCA in Thy1-SNCA mouse brains after ASO^A19^ treatment. Immunohistological images of various brain areas in wild type, control, and ASO^A19^-treated Thy-1 SNCA mice using a monoclonal antibody that recognizes phosphorylated SNCA. ASO^A19^-treated Thy1-SNCA mice showed partial reduction of phosphorylated SNCA in the cortex, olfactory bulb, and dentate gyrus relative to control mice (arrow). Scale bars: 100 μm.

In this mouse model, we quantified expression levels of hSNCA protein extracted from left cerebral hemisphere by enzyme-linked immunosorbent assay (ELISA), which is quantitatively sensitive to detect changes in the level of highly expressed hSNCA protein by the Thy1 promoter. We found that hSNCA protein appeared to partition primarily into the Triton X-100 soluble fraction, in contrast to the 10-fold smaller fraction of hSNCA protein in the insoluble fraction. ELISA showed that the levels of SNCA protein in ASO^A19^-treated mice were significantly decreased in both the soluble and insoluble fractions (soluble hSNCA protein in ASO-injected mice/control = 87.3% ± 2.9%, p = 0.006, t-test and insoluble hSNCA protein in ASO-injected mice/control = 81.5% ± 3.6%, p = 0.0073, t-test), indicating that ASO^A19^ efficiently reduced hSNCA protein levels in the brains of Thy-1 SNCA mice **(**Supplemental Fig. 5**)**. Histological evaluation of brain sections from wild type, PBS-, and ASO^A19^-injected Thy-1 SNCA mice four weeks after treatment with an antibody specific to hSNCA further confirmed SNCA protein knockdown in ASO^A19^-injected mice of the 3 groups **(**Supplemental Fig. 6**)**. In addition, to assess the effect of SNCA knockdown on the formation of inclusions, we immunostained the brain sections for phosphorylated SNCA, which is frequently used as an indicator for SNCA inclusions. Partial decrease of phosphorylated SNCA density was seen in the cortex, dentate gyrus, and olfactory bulb of ASO^A19^-injected Thy-1 SNCA mice **(**Fig. 7**)**.

**Figure 8:**
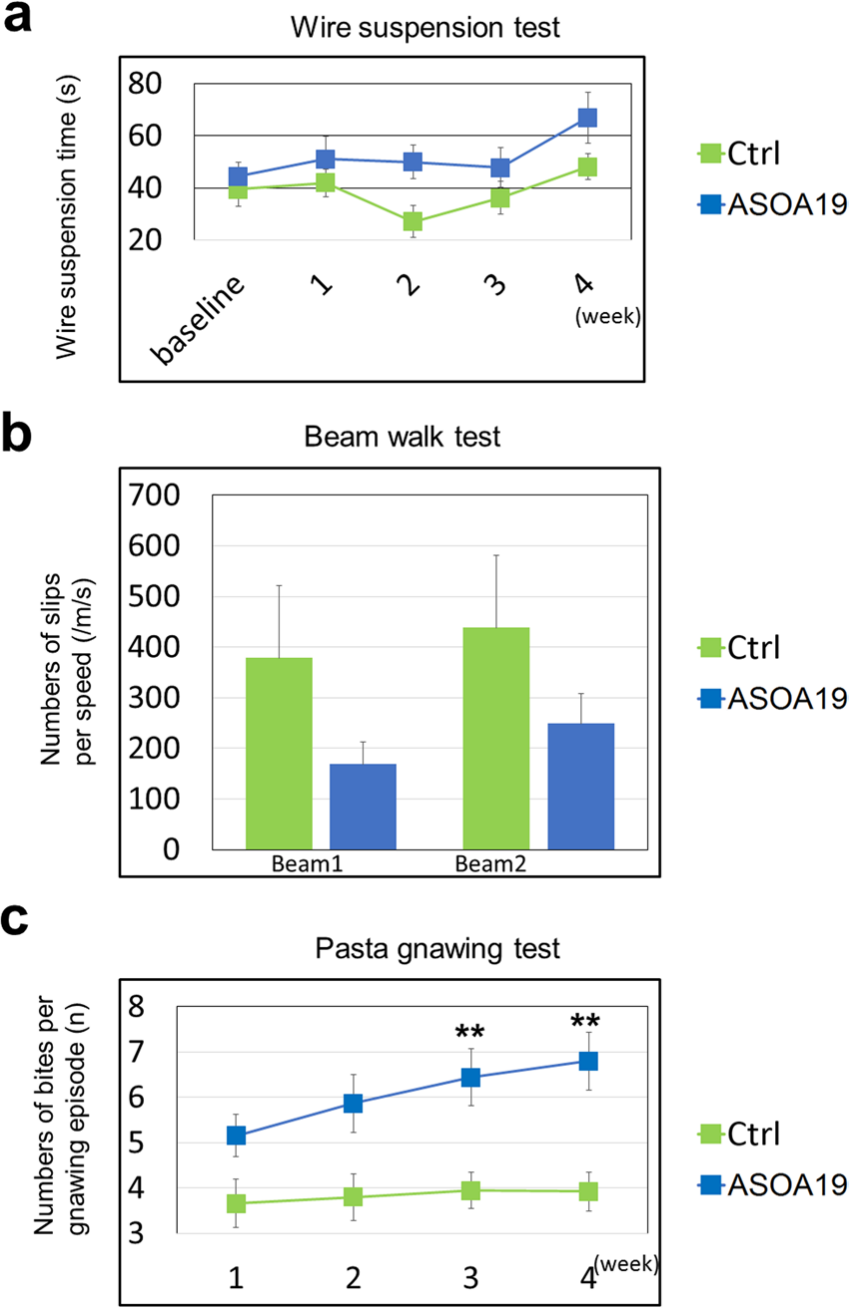
Intracerebroventricular administration of ASO^A19^ improved motor function in Thy1-SNCA mice. (**a**) Hanging time for the wire suspension test, which was performed every seven days after treatment with ASO^A19^ or control. ASO^A19^-treated mice showed a trend of longer suspension compared with controls. Data are expressed as mean ± SEM (n = 17 ASO^A19^ and 13 control). (**b**) The beam walking test was performed four weeks after administration of ASO^A19^ or control. The graph shows slips per speed. ASO^A19^-treated mice showed a trend of fewer slips than controls. Data are expressed as mean ± SEM (n = 17 ASO^A19^ and 13 control). (**c**) Pasta gnawing test showing the number of bites per gnawing episode every seven days after administration of ASO^A19^ or control. ASO^A19^-treated mice showed an increased number of bites, indicating improvement in hypokinetic defects. Data are expressed as mean ± SEM (n = 17 ASO^A19^ and 13 control). **p < 0.01 by t-test.

Thy-1 SNCA mice also show significant impairments in motor performance at the age of two months, which can be reversed by the dopamine precursor L-dopa, similar to PARK4 and other PD patients^29–31^. To determine if ASO^A19^ could ameliorate behavioral defects in Thy1-SNCA mice, we examined motor behavior in control and ASO^A19^-injected mice using the wire suspension test every seven days after injection (See Material and Method). We found that mice injected with ASO^A19^ showed better performance than control Thy1-SNCA mice. The mean test time for ASO^A19^-injected mice was 67.1 s on day 27 after injection, whereas the mean time was 48.2 s for control mice **(**Fig. 8a**)**. In addition, mice injected with ASO^A19^ tended to show improvement in the beam walk test, which was performed 28 days after injection using two beams with different widths. We measured the frequency of slips from the beam and normalized this value to the walking speed along the beams. ASO^A19^-injected mice showed tendency of reduced frequency of slips per speed compared to controls (ASO^A19^ vs. control: 168.7 vs 378.8 per m/s (beam 1) and 248.8 vs. 438.1 per m/s (beam 2), Fig. 8b). Lastly, we examined the effects of ASO^A19^ on the pasta gnawing test, which can sensitively evaluate eating behavior that requires fine coordination of limb and oromotor functions^32–34^. Notably, ASO^A19^-injected mice showed significantly better performances than control mice. On day 20 after injection, the mean number of bites per episode was 3.94 for control mice and 6.44 for ASO^A19^-injected mice (p = 0.0051, t-test). On day 27 after injection, the mean number of bites per episode was 3.92 for control mice and 6.8 for ASO^A19^-injected mice (p = 0.0021, t-test) **(**Fig. 8c**)**. Taken together, these data indicate that ASO^A19^ can improve behavioral defects observed in Thy1-SNCA mice accompanied by downregulation of SNCA.

## Discussion

In the present study, we generated ASO^A19^ capable of reducing the level of hSNCA mRNA in HEK293 cells and found that ASO^A19^ is superior to ASO^L19^ for decreasing hSNCA mRNA. Further, we confirmed that ASO^A19^ was widely distributed in the CNS following a single intracerebroventricular administration. This study is the first to report that intracerebroventricular administration of ASO in the absence of any carrier or conjugation can knockdown the level of hSNCA and improve motor dysfunction in transgenic animal PD models.

SNCA protein is one of the most promising therapeutic targets for the treatment of PD^35^. We showed that ASO^A^^19^ can target wild type and mutant hSNCA, indicating its applicability for both familial and sporadic PD. Moreover, reducing the level of SNCA expression is likely to protect against neuronal toxicities. We previously reported that reduction of SNCA expression enabled the survival of more TH-positive neurons in 1-methyl-4-phenyl-1,2,3,6-tetrahydropyridine model rats^36^. SNCA knockdown by shRNA attenuates the progression of motor deficits in rotenone-exposed rats^37^, suggesting that reduction of SNCA expression might also be beneficial for PD. Benskey *et al*. reported that excessive reduction of SNCA within nigrostriatal neurons induced rapid up-regulation of the major histocompatibility complex class 1 (MHC-1) and resulted in the death of affected neurons^38^. However, several studies have shown that normalization of overexpressed SNCA decreased neurodegeneration of dopaminergic neurons and improved motor function in animal models^37,39^. These data imply that adequate downregulation of SNCA can prevent neurodegeneration with little disadvantages.

To improve the efficacy of antisense oligonucleotides for *in vivo* therapeutic application, we developed a less toxic ASO containing AmNA^25,26^. We then applied AmNA-ASO targeting Y-box binding protein-1 effectively as an antiangiogenic cancer therapy^27^. To increase ASO stability *in vivo* and reduce the number of treatments, we applied the technique of AmNA modification in ASO targeting hSNCA. Importantly, AmNA modification enhanced the potency of ASO for targeting SNCA **(**Fig. 2a–d**)**. Consistently, single intracerebroventricular administration of ASO^A19^ was effective for targeting SNCA in the mouse CNS even two weeks after injection **(**Fig. 6c,d**)**, supporting the stability of intracerebroventricularly administered ASO^A19^ in patients with α–synucleinopathies.

In an alternative approach to targeting SNCA protein, antibodies against the C-terminus of SNCA protein were shown to reduce accumulation of C-terminal truncated SNCA protein, prevent TH loss in the striatum, and improve motor deficits in SNCA transgenic mouse models^40^. A phase I study of administration of this anti-SNCA antibody, PRX002, was performed and showed that serum SNCA protein could be modulated without any serious adverse events^41^. However, this anti-SNCA immunotherapy works primarily by recognizing and clearing extracellular SNCA protein to block cell-to-cell propagation of SNCA in the brain and is less effective at targeting intraneuronal SNCA. ASO^A19^ administration could be a complementary approach used with anti-SNCA immunotherapy.

Although we found no off-target or toxic effects of ASO^A19^ **(**Fig. 6b) (Supplemental Fig. 7**)**, the safety of ASO^A19^ injection must be confirmed for future clinical applications. Studies using SNCA knock-out mice showed that SNCA might be required for synaptic function^42,43^ and maintenance of subependymal neural stem cells^44^. It is important to note that antisense therapy does not cause total loss of SNCA expression **(**Fig. 6a**)**. Hence, a modest reduction of SNCA expression is not likely to cause serious defects, even if SNCA is required for some biological functions in the human brain.

In conclusion, we demonstrated that intracerebroventricular administration of AmNA-ASO can reduce SNCA mRNA and the corresponding protein levels, resulting in improvement of some motor dysfunctions observed in PD model mice. Remarkably, intracerebroventricular administration of nusinersen, an ASO for treating SMA, was recently approved^19–22^. Our findings highlight the potential of AmNA-ASO as a novel therapy for PD and other synucleinopathies.

## Material and Methods

### Oligonucleotides

The ASOs used in this study, which were synthesized by GeneDesign, are gapmer-type 13-15-mer phosphorothioate oligonucleotides containing five AmNA modifications **(**Supplemental Fig. 1b**)**.

AmNA-ASOs containing specific DNA sequences (TCC and TGC), which have been shown to bind to hepatocellular proteins and cause hepatotoxicity^45^, induce cellular responses mediated by Toll-like receptor 9, and cause strong inflammatory responses (CpG), were excluded^46^. The loop structure of SNCA mRNA, where ASOs easily bind target RNA, was predicted using the mFold web server^47^ and the 50 most effective sequences targeting hSNCA mRNA were selected.

### Cell culture and transfection

HEK293 cells (ATCC CRL-1573) were cultured in Dulbecco’s Modified Eagle Medium (Sigma) supplemented with 10% fetal bovine serum at 37 °C in a humidified chamber of 95% O_2_ and 5% CO_2_. Cells were plated into 12-well plates 24 hours before transfection. AmNA-ASOs were transfected into HEK293 cells with Lipofectamine 2000 (Life Technologies) according to the manufacturer’s protocols. The final concentration of AmNA-ASOs was adjusted to 50 nM. All ASOs were tested in triplicate.

### Quantitative RT-PCR of total RNA extracted from HEK293 cells and mouse brains

Total RNA was extracted from HEK293 cells using a RNeasy Mini kit (QIAGEN). RNA was reverse transcribed to complementary DNA (cDNA) using the Superscript III First Strand cDNA system (Life Technologies) according to the manufacturer’s protocol and cDNA was used as the template for qPCR. qPCR was performed using TaqMan Gene Expression assays on an ABI PRISM 7900HT Sequence Detection System (Life Technologies). Relative mRNA expression was normalized by the 18 s ribosomal RNA level in each sample and calculated using the delta-delta Ct method. Taqman primer/probe sets specific for hSNCA, hSNCB, hSNCG, mouse Snca, mouse Sncb, mouse Sncg, and 18 s ribosomal RNA were purchased from Thermo Fisher Scientific.

To quantify hSNCA mRNA levels in mice, brain samples were collected and separated into left and right cerebral hemispheres. Left hemispheres were used for qPCR and right hemispheres for Western blot analysis. Left hemispheres were immediately snap-frozen in liquid nitrogen and stored at −80 °C. For analysis, frozen left brain hemispheres were ground using a prechilled mortar and pestle with liquid nitrogen occasionally added to prevent thawing. After the tissue was finely ground, ISOGEN (Nippon gene) was added to isolate total RNA, according to the manufacturer’s instructions. Reverse transcription and qPCR were performed as described above.

### Western blot analysis of SNCA protein from HEK293 cells and mouse brains

Proteins were extracted from HEK293 cells using the CelLytic M Cell Lysis Reagent (Sigma-Aldrich) with protease and phosphatase inhibitor cocktail. Protein concentration was measured using a Pierce™ BCA Protein Assay Kit (Thermo Fisher Scientific). Cell lysates were mixed with Laemlli sample buffer, boiled, and separated on polyacrylamide gel. After protein transfer to polyvinylidene fluoride membranes, the blots were probed with the primary antibodies, α-synuclein (BD Transduction Laboratories, cat #610787, 1:10000 dilution) and actin (Millipore, cat #MAB1501, 1:1000 dilution); visualized with secondary antibodies, HRP-conjugated anti-mouse IgG or anti-rabbit IgG (GE Healthcare); and developed using ECL Prime Western Blotting Detection Reagent (GE Healthcare). The signals of immunoblots were acquired with a Chemidoc Touch Imaging System (Bio-Rad).

To quantify hSNCA protein in mouse brains, proteins were extracted from the right cerebral hemispheres using the CelLytic™ MT Cell Lysis Reagent for mammalian tissues (Sigma-Aldrich) with protease and phosphatase inhibitor cocktail. The BCA assay and Western blotting were performed as described above. The primary antibody used for hSNCA detection was α-synuclein (211) (Santa Cruz, cat #sc-12767, 1:1000 dilution).

### Mouse strains

TH-SNCA-140 m transgenic mice with a C57BL/6J background carrying the hSNCA gene with a Ala53Thr mutation, for which expression is driven by the TH promoter^28^, and Thy1-SNCA transgenic mice (Line 61) with a C57BL/6 × DBA/2F1 background carrying human wild type SNCA driven by the murine thymus cell antigen 1 promoter^29–31^ were used.

### Animal housing

Mice were housed in individual ventilated cages. Each cage contained a maximum of four mice of the same sex. Mice were housed under a 12 hours light/dark cycle and standard housing conditions with ad libitum access to food and water for one week before the experiment. All animal experiments were conducted according to ARRIVE guidelines.

### Intracerebroventricular injection of AmNA-ASOs into mice

Mice were anesthetized with an intraperitoneal injection of cocktails comprising medetomidine (2 mg/kg), midazolam (10 mg/kg), butorphanol (1 mg/kg) or ketamine (50 mg/kg), medetomidine (0.3 mg/kg), and butorphanol (5 mg/kg). Anesthetized mice were placed into a stereotaxic frame and a single burr hole was drilled to allow placement of the stereotactic needle (Hamilton®). The coordinates for injection were 0.3 mm anterior and 1.0 mm lateral to Bregma and 3 mm below the dura. AmNA-ASOs dissolved in 10 μl phosphate-buffered saline (PBS) were injected into the left lateral ventricle at a rate of 2 μl/min^48^.

### Behavioral testing

For mice administered with AmNA-ASOs, body weight was measured and pasta gnawing test^32^ and wire suspension test^49^ were performed four times per week. The beam walk test^50,51^ was performed once after the final pasta gnawing and wire suspension tests. For the pasta gnawing test, mice were brought into the experimental room at least two hours before testing. The cage top, water bottle, and food pellets were removed (only the cage lid remained) and a small piece of dry spaghetti (approximately 5 mm) was placed in the cage. After habituation, the home cage was placed on the testing table. Spaghetti noodles were broken into several pieces approximately 1 cm long and placed in the center of the cage. A microphone was placed above the noodle pieces and recording was initiated as soon as the animal started to eat. The number of bites per gnawing episode and biting frequency were evaluated. The acquired gnawing pattern data were analyzed using Avisoft SASLab Pro.

For the wire suspension test, a wire cage lid was used with duct tape placed around the perimeter to prevent the mouse from walking off the edge. The animal was placed on top of the cage lid and the lid was lightly shaken three times to force the mouse to grip the wires. The lid was then turned upside down and held approximately 50–60 cm above a soft underlay, high enough to prevent the mouse from jumping down but not high enough to cause harm in the event of a fall. The latency to fall was quantified.

For the beam walk test, we used long strips of wood (beams) with a 1000 × 13 or 10 mm cross-section. The beams were placed horizontally 50 cm above the surface, with one end mounted on a narrow support and the other end attached to the home cage into which the mouse could escape. A desk lamp was positioned above the start of the beam. Three training trials were performed prior to testing using three different starting points on the 20 mm square beam: close proximity to the home cage (trial 1), the center of the beam (trial 2), and at the brightly illuminated end of the beam (trial 3). After the mice were trained, they underwent test trials on each of the square beams. The test trials were videotaped and then evaluated with Observer XT 10.5 (Noldus). The latency to traverse each beam and the number of times the hind feet slipped off each beam were recorded for each trial.

### Immunofluorescence analysis

Frozen brain hemispheres from non-injected and Alexa 488-conjugated AmNa-injected C57BL/6 mice were cryosectioned coronally at 30 µm thickness on a Leica CM1850. Sections were transferred to 30% sucrose solution in PBS (Thermo Scientific) and stored at 4 °C. Immunofluorescence experiments were performed using the following protocol: sections were washed 3 × 5 min in PBS, fixed with 4% PFA in PBS for 10 min, washed 3 × 5 min in PBS with 0.3% Triton X-100 (PBST), blocked 1 hour with 10% Block Ace PBS, incubated with primary antibodies in 10% Block Ace PBS overnight at 4 °C, washed 3 × 5 min in PBST, incubated with secondary antibodies in PBST for 1 h at room temperature (RT), washed 3 × 5 min in PBST, and mounted onto regular glass slides (Platinum Pro, Matsunami) with mounting medium with DAPI (Vectashield). After immunostaining, the mounted sections were observed and imaged under a confocal laser-scanning microscope (FV1200, Olympus). Primary antibodies against TH (Calbiochem, cat#657012, 1:1000 dilution), NeuN (Millipore, cat #MAB377, 1:1000 dilution), MAP2 (Sigma, cat # M4403, 1:200 dilution), GFAP (Dako, cat #Z0334, 1:1000 dilution), O4 (Chemicon international, cat #MAB345, 1:200 dilution), and Iba1 (Wako, cat #015-25191, 1:1000 dilution) were used. As secondary antibodies, donkey anti-mouse and donkey anti-rabbit labeled with Cy3 (Jackson ImmunoResearch) were used.

### Histological analysis of SNCA protein in mouse brains

Brain samples were taken from PBS- and AmNa-ASO-treated Line 61 mice and wild type littermates. The hemispheres were divided at the midline. One hemisphere was fixed by immersion in 4% paraformaldehyde in 0.1 M phosphate buffer pH 7.4 for 2 hours at RT, cryoprotected in 15% sucrose in PBS overnight, embedded in OCT medium in cryomolds, and snap-frozen in dry ice-cooled liquid isopentane. Frozen samples were stored at −80 °C until sectioning. Frozen brain hemispheres were cryosectioned sagittally at 30 µm thickness on a Leica CM1850. Sections were transferred to 30% sucrose solution in PBS (Thermo Scientific) and stored at 4 °C. For immunostaining, sections were washed and incubated with primary and secondary antibodies as previously described in Furuya *et al*.^52^. In brief, the following primary antibodies were used: phosphorylated α-synuclein antibody, a mouse monoclonal antibody against α-synuclein phosphorylated at Ser129 (Wako, cat # 015-25191, 1:1000 dilution) and Syn211 (Invitrogen, AHB0261, 1:1000 dilution), and a mouse monoclonal antibody that specifically recognizes human α-synuclein. After immunostaining, mounted sections were observed and imaged under a fluorescence microscope (BZ-X700, Keyence).

### ELISA for SNCA protein expression

Mouse brains were collected 30 days after injection. Mashed left brain hemispheres were homogenized in lysis buffer (20 mM Tris-HCl, pH 7.4, 50 mM NaCl, 1% Triton X-100, 0.2 mM Sodium-orthovanadate, protease inhibitor cocktail (Calbiochem), and phosphatase inhibitor cocktail (Sigma)). Homogenates were incubated for 30 min on ice, followed by centrifugation at 15,000 g for 60 min at 4 °C. Supernatant was collected as the Triton X-100 soluble fraction. The Triton X-100-insoluble pellet was washed once in lysis buffer and then dissolved in lysis buffer containing 2% SDS. The resulting homogenate in 2% SDS was collected as the Triton X-100 insoluble fraction. hSNCA protein levels in the Triton X-100 soluble and insoluble fractions of all mice were determined using a commercially-available immunosorbent assay (Meso Scale Discovery, Cat#. K151TGD-4; Lot# K00E0147) according to the manufacturer’s protocol. Plates were read on the Sector Imager 2400. For the assay, Triton X-100 soluble fractions were diluted 1:20000 and insoluble fractions 1:2000 in assay buffer. SNCA protein levels were evaluated in comparison to an adequate peptide standard as μg hSNCA and normalized to tissue weight (wet weight).

### Statistical analysis

Dunnett’s test was used to compare several treatments with a single control (e.g. Fig. 1a). One-way ANOVA, Tukey post hoc test was performed to compare the efficacy of control, ASO^A19^, and ASO^L19^ in HEK293 cells. Two-tailed unpaired t-tests were performed to compare the efficacy of control vs ASO^A19^ in mice. For all figures, the error bars represent the standard error of the mean (SEM). P-values below 0.05 were considered statistically significant.

### Study approval

This research was approved by the institutional ethics committee. All of the procedures for animal experiments were approved by the institutional animal care and use committee of Osaka University.

## Supplementary information

Dataset 1
