# Supplementary material for: Amido-bridged nucleic acid (AmNA)-modified antisense oligonucleotides targeting α-synuclein as a novel therapy for Parkinson’s disease
Source: Sci Rep. 2019 May 21;9:7567. doi: 10.1038/s41598-019-43772-9 (PMC6527855; doi:10.1038/s41598-019-43772-9)
Supplement: Supplementary file 1 — Dataset 1 [file 41598_2019_43772_MOESM1_ESM.pdf]

Amido-bridged nucleic acid (AmNA)-modified antisense oligonucleotides targeting α-synuclein as a novel therapy for Parkinson's disease

Takuya Uehara, Chi-Jing Choong, Masayuki Nakamori, Hideki Hayakawa, Kumiko Nishiyama, Yuuya Kasahara, Kousuke Baba, Tetsuya Nagata, Takanori Yokota, Hiroshi Tsuda, Satoshi Obika, Hideki Mochizuki

Supplemental data

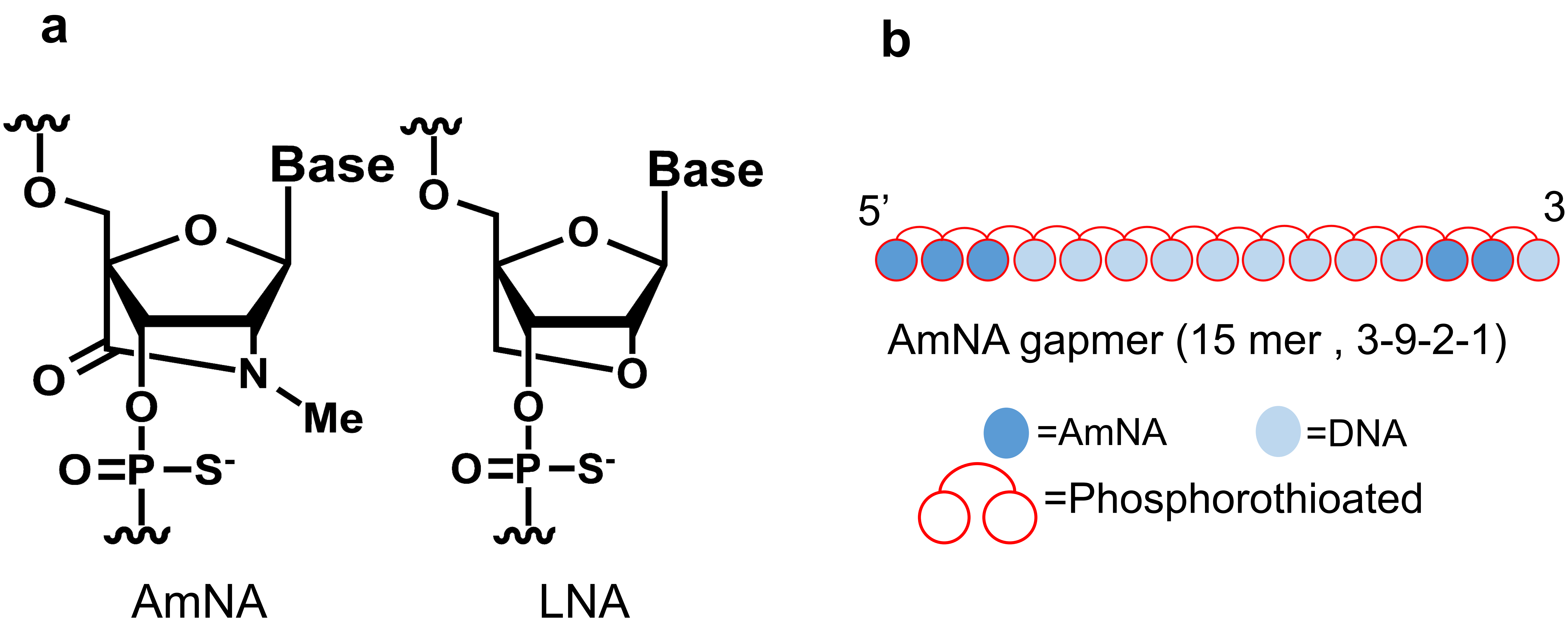

Supplemental Figure 1. Design of gapmer-type ASO containing AmNA

(a) Chemical structure of AmNA and LNA (2',4' -BNA) phosphorothioated monomer. AmNA nucleotide is modified with an amide bond bridged between 2' and 4'carbon of the ribose, whereas LNA nucleotide is modified with a bridge connecting the 2' oxygen and 4' carbon of the ribose.

(b) Diagram of a 15-mer AmNA gapmer, which contains AmNA at each end flanking the central bases of DNA in the motif of 3AmNA-9DNA-2AmNA-1DNA (3-9-2-1). Nucleotides are composed of AmNA and DNA that have complementary sequence to their target human SNCA mRNA.

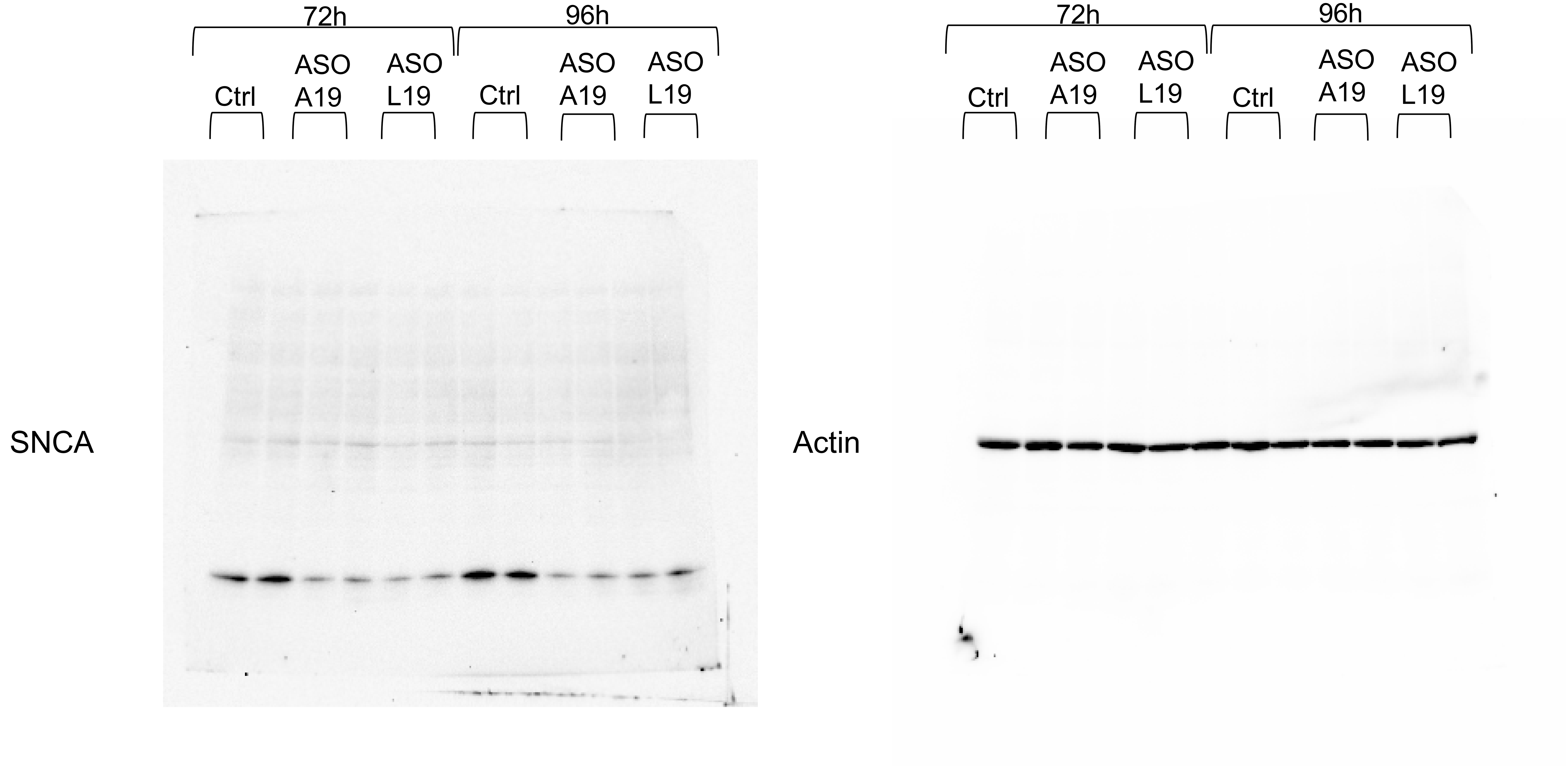

Supplemental Figure 2. Full-length gels of immunoblotting

Immunoblotting with anti-SNCA and anti-beta-actin as internal control. Proteins were extracted from HEK293 cells 72 and 96 hours after transfection of ASO^{A19} and ASO^{L19}. Immunoblot and densitometry analysis show both ASO^{A19} and ASO^{L19} reduced the levels of SNCA protein. Cropped images were shown in **Figure 2c.**

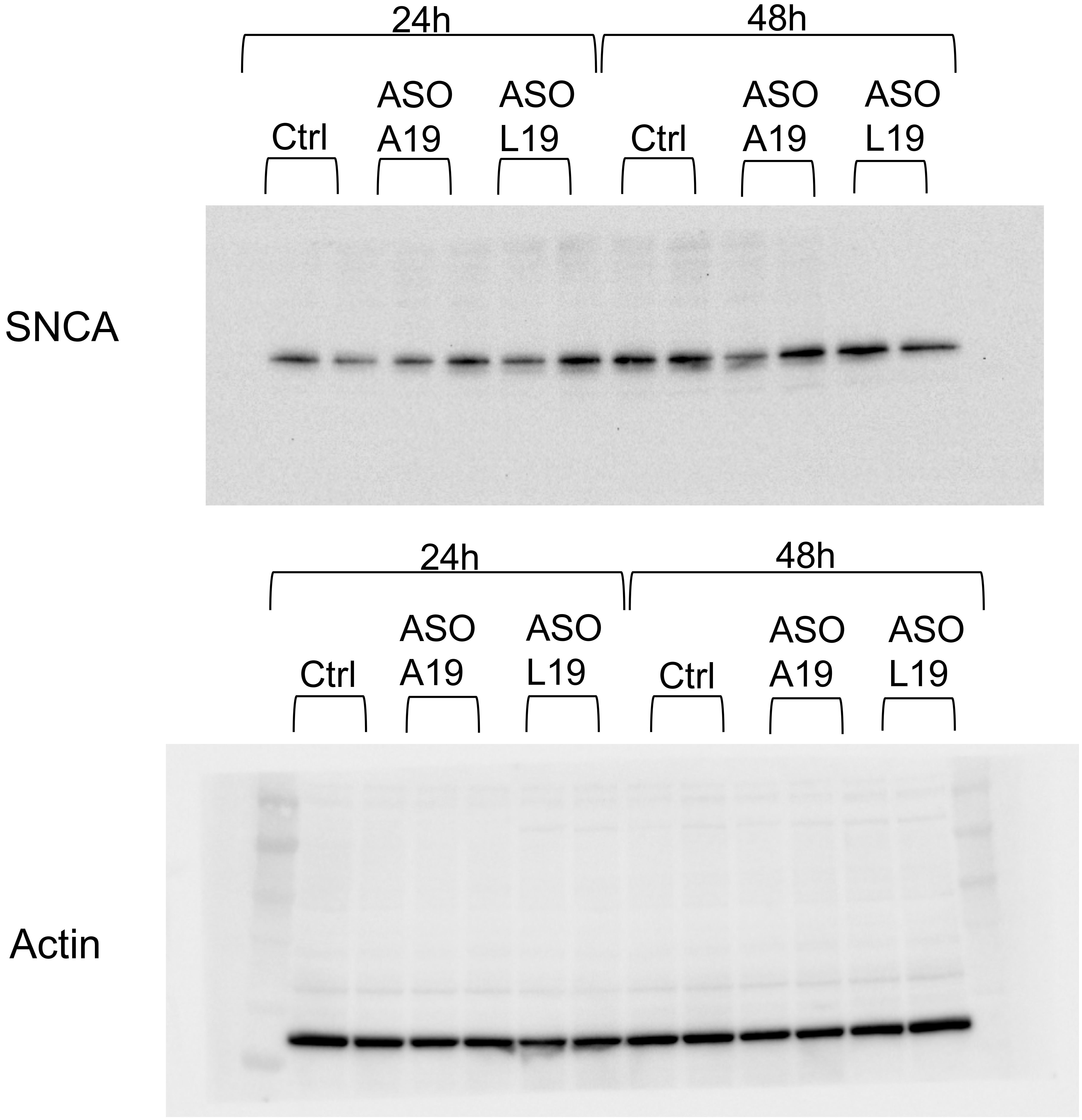

Supplemental Figure 3. Full-length gels of immunoblotting

Immunoblotting with anti-SNCA and anti-beta-actin as internal control. Proteins were extracted from HEK293 cells 24 and 48 hours after transfection of ASO^{A19} and ASO^{L19}. No significant downregulation of SNCA protein by ASO^{A19} and ASO^{L19} was observed at these time points.

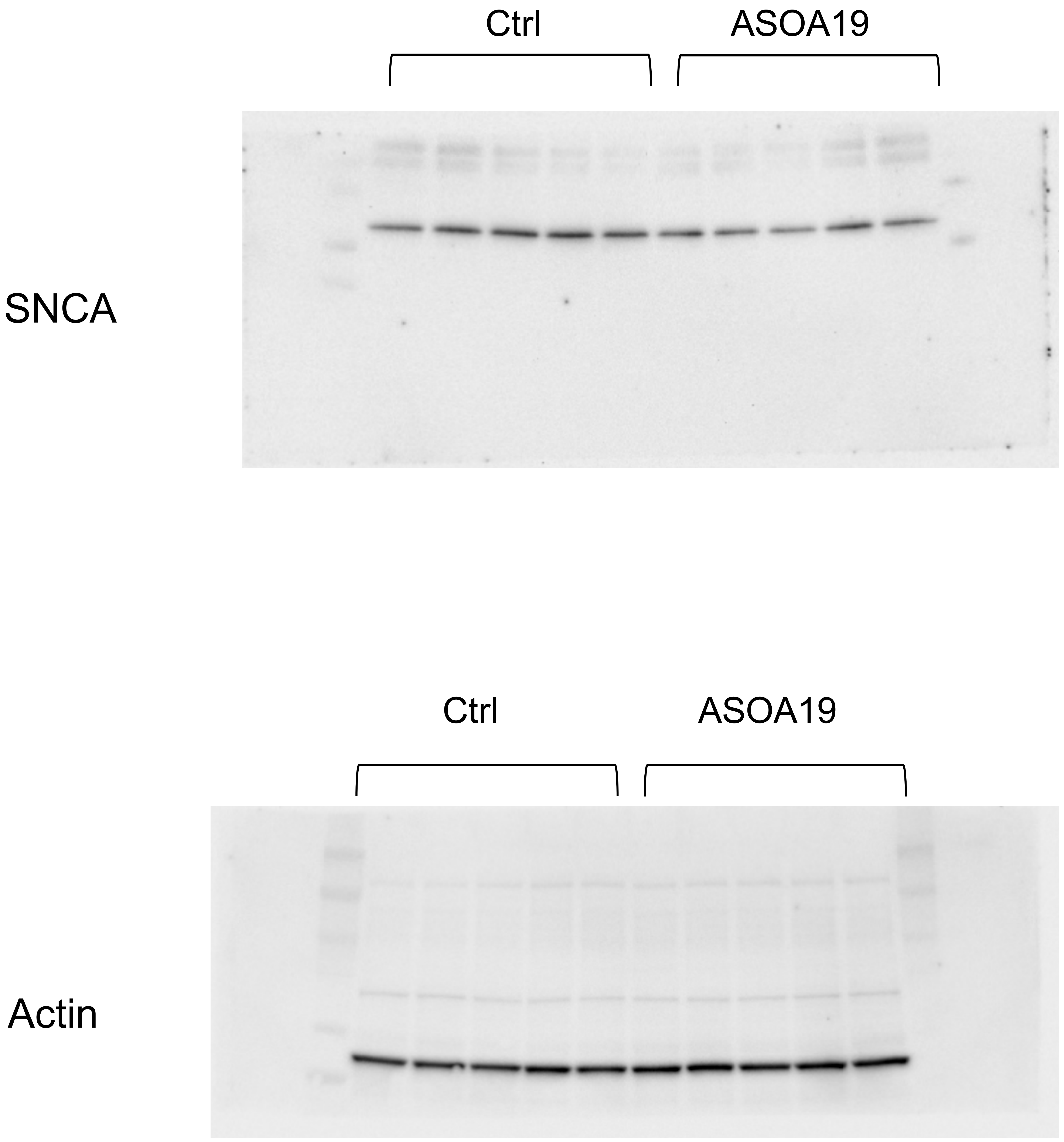

Supplemental Figure 4. Full-length gels of immunoblotting

Immunoblot and densitometry analysis showing ASO^{A19} reduced the levels of SNCA protein. Protein was extracted from right cerebral hemisphere two weeks after administration of ASO^{A19} and subjected to immunoblot. Cropped images were shown in **Figure 6.**

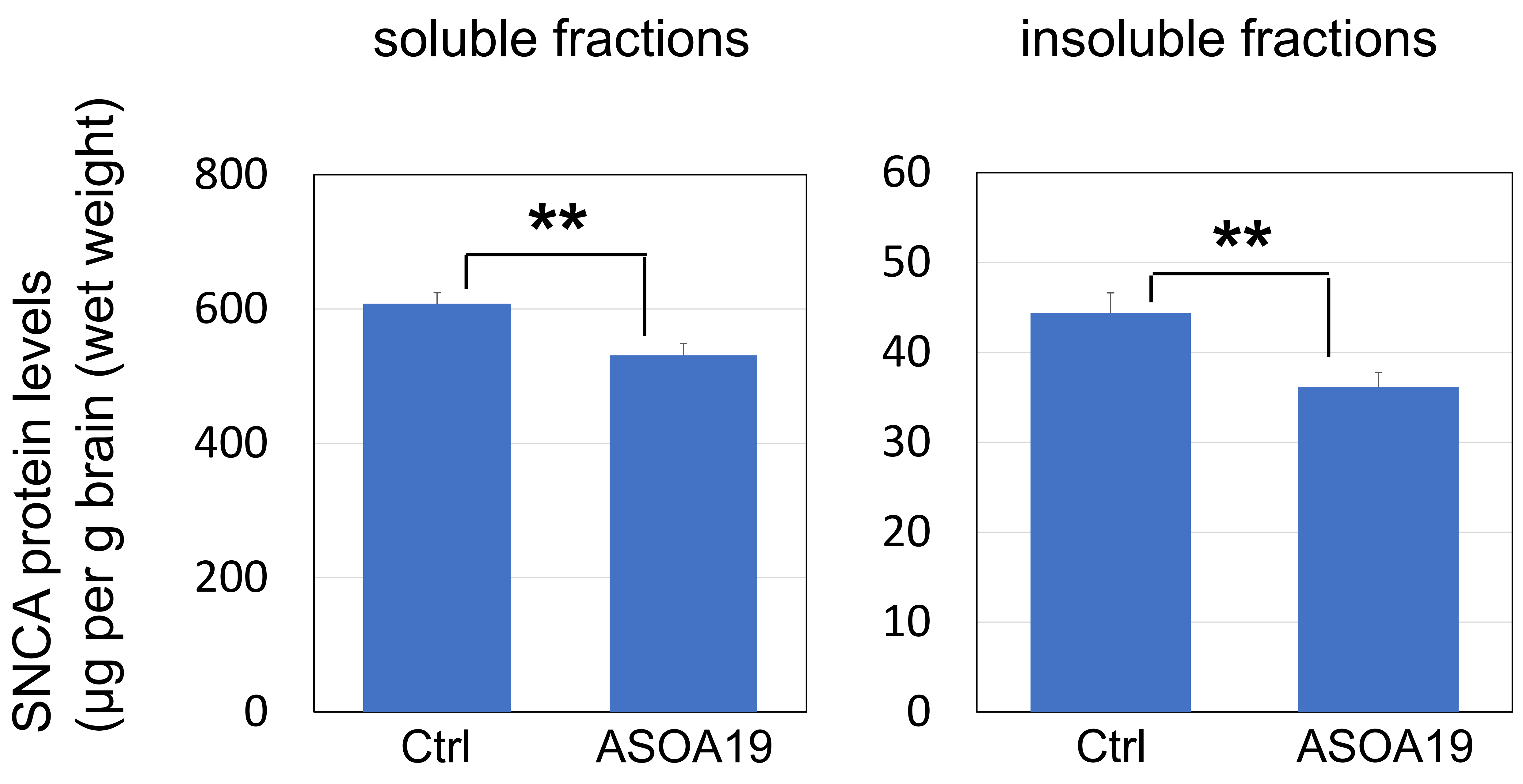

Supplemental Figure 5. Intracerebroventricular administration of ASO^{A19} reduces the levels of SNCA protein in the brain of Thy-1 SNCA mice

ELISA analysis of the levels of SNCA protein extracted from left cerebral hemisphere of Thy1-SNCA mice. Protein was extracted 28 days after ASO^{A19} or control injection and separated into detergent-soluble and insoluble fractions. ASO^{A19} significantly reduced the levels of SNCA protein both in the detergent-soluble and insoluble fractions (soluble and insoluble). Data expressed as mean±SEM (n=17 (ASO^{A19}) and 13 (control)). **$p<0.01$ by t-test.

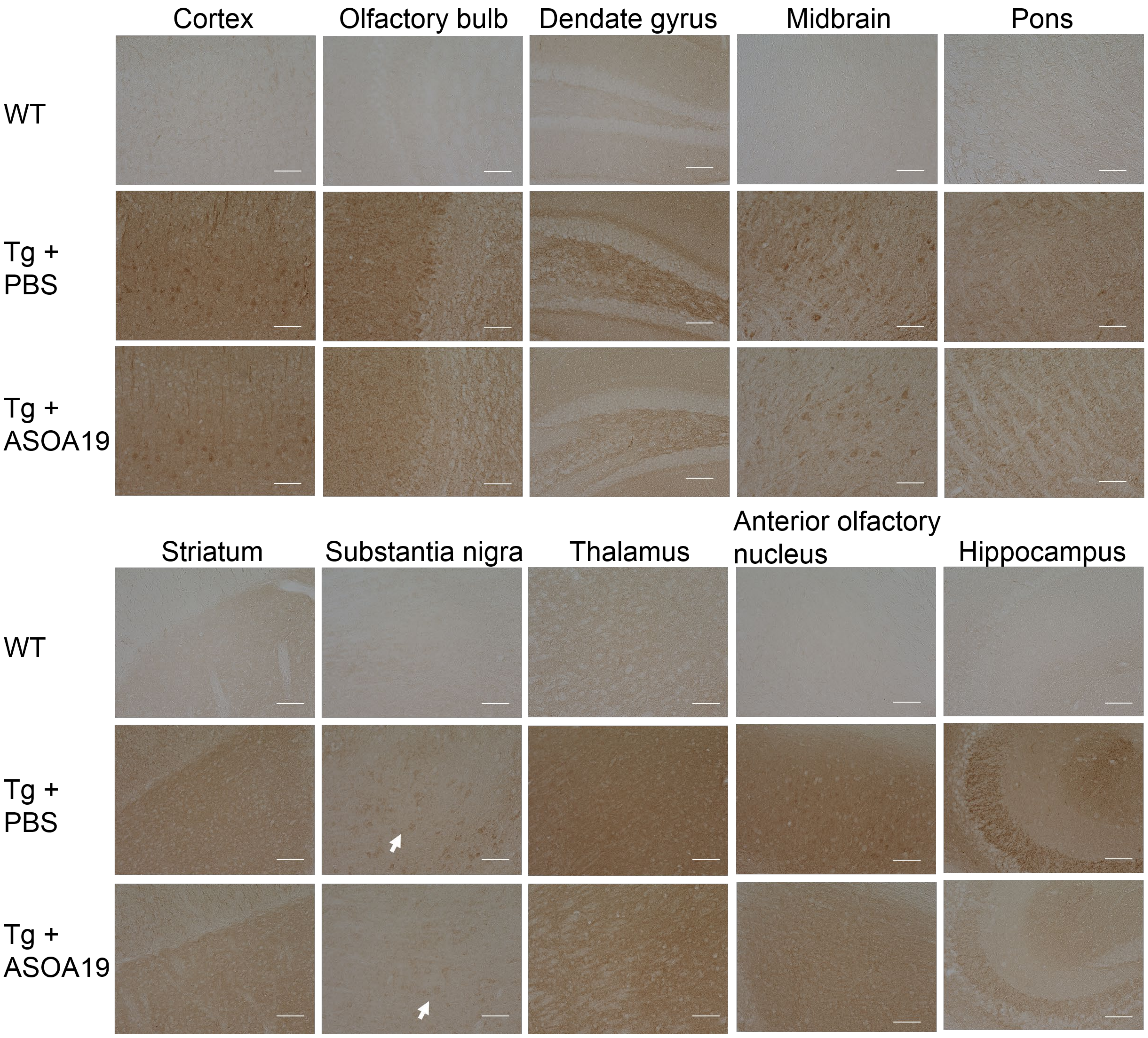

Supplemental Figure 6. Decrease of hSNCA in the brain of Thy1-SNCA mice after ASO^{A19} treatment

Expression of hSNCA in the brain of wild-type, control and ASO^{A19}-treated Thy-1 SNCA mice. Representative images of brain sections immunostained with a monoclonal antibody that specifically recognizes hSNCA are shown. ASO^{A19}-treated Thy1-SNCA mice show milder staining compared with control mice, indicating downregulation of hSNCA levels in brain. Scale bars: 100 µm.

a

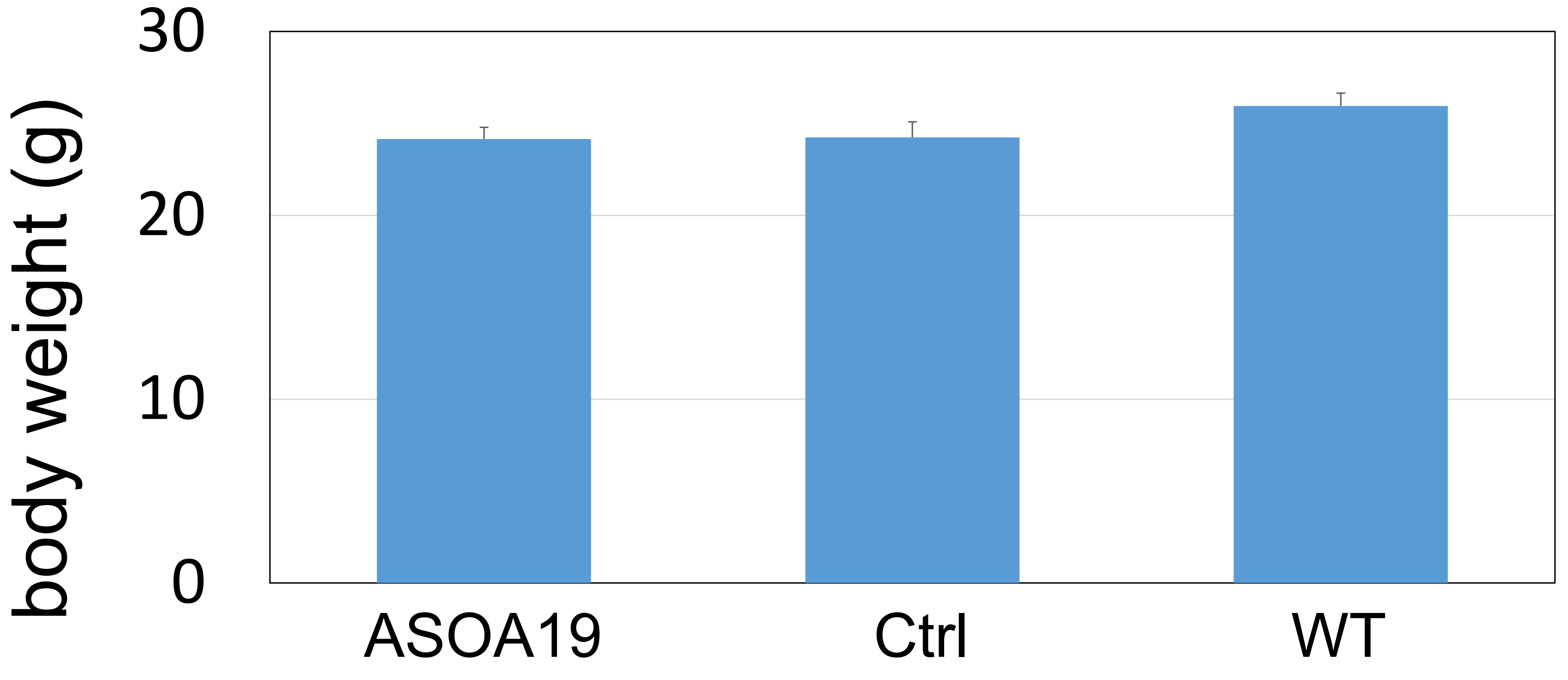

b

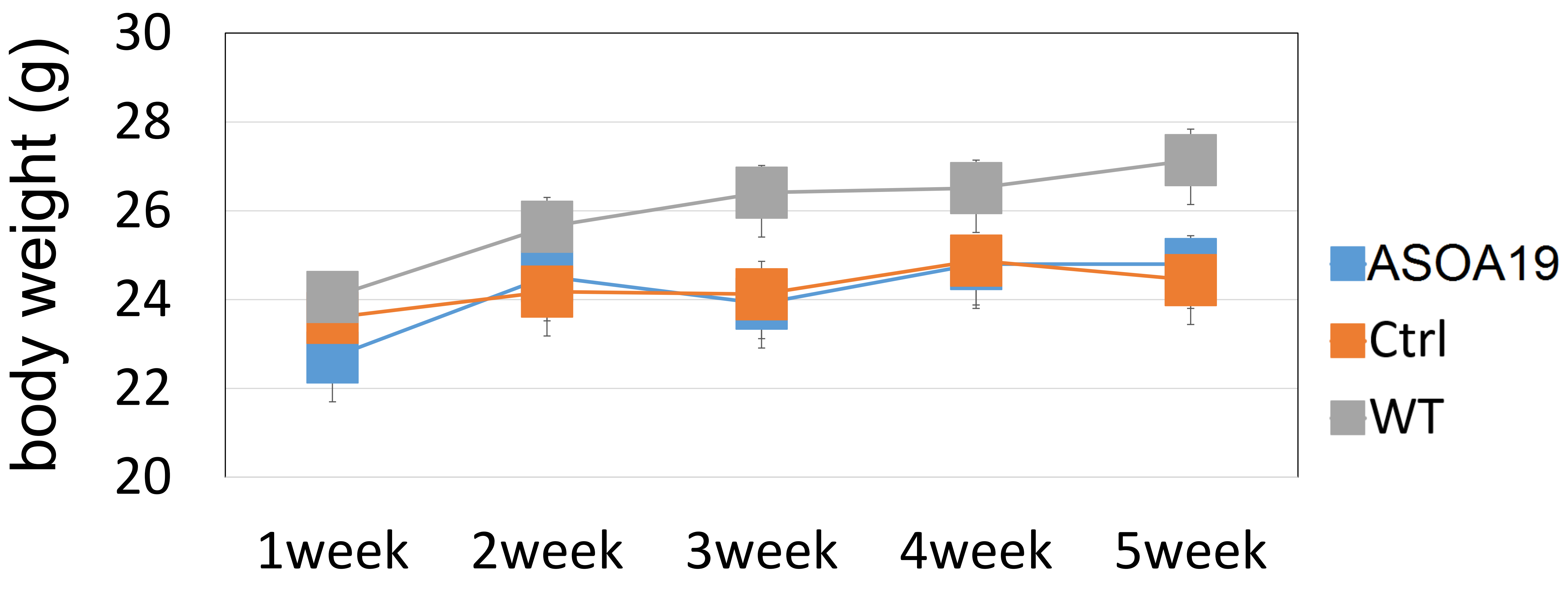

Supplemental Figure 7. mouse body weight after ASO^{A19} treatment

(a) Mean body weight of each treatment group, ASO^{A19}-treated Thy-1 SNCA mice (ASOA19), PBS-treated Thy-1 SNCA mice(Ctrl), and wild type mice (WT), averaged over five time points (one per weeks). Data are displayed as means±SEM

(b) Body weight development over time. *$p<0.05$ by Bonferroni post test (PBS vs WT).
